# Spread and clinical severity of respiratory syncytial virus A genotype ON1 in Germany, 2011–2017

**DOI:** 10.1186/s12879-019-4266-y

**Published:** 2019-07-12

**Authors:** Andrea Streng, David Goettler, Miriam Haerlein, Lisa Lehmann, Kristina Ulrich, Christiane Prifert, Christine Krempl, Benedikt Weißbrich, Johannes G. Liese

**Affiliations:** 1 Department of Pediatrics, University Hospital of Würzburg, Josef-Schneider-Str. 2, D-97080 Würzburg, Germany; 2 Institute for Virology and Immunobiology, University of Würzburg, Würzburg, Germany

**Keywords:** Children, Respiratory tract infection, RSV-A ON1, Epidemiology, Disease severity

## Abstract

**Background:**

The Respiratory Syncytial Virus (RSV) A genotype ON1, which was first detected in Ontario (Canada) in 2010/11, appeared in Germany in 2011/12. Preliminary observations suggested a higher clinical severity in children infected with this new genotype. We investigated spread and disease severity of RSV-A ON1 in pediatric in- and outpatient settings.

**Methods:**

During 2010/11 to 2016/17, clinical characteristics and respiratory samples from children with acute respiratory tract infections (RTI) were obtained from ongoing surveillance studies in 33 pediatric practices (PP), one pediatric hospital ward (PW) and 23 pediatric intensive care units (PICU) in Germany. RSV was detected in the respiratory samples by PCR; genotypes were identified by sequencing. Within each setting, clinical severity markers were compared between RSV-A ON1 and RSV-A non-ON1 genotypes.

**Results:**

A total of 603 children with RSV-RTI were included (132 children in PP, 288 in PW, and 183 in PICU). Of these children, 341 (56.6%) were infected with RSV-A, 235 (39.0%) with RSV-B, and one child (0.2%) with both RSV-A and RSV-B; in 26 (4.3%) children, the subtype could not be identified. In the 341 RSV-A positive samples, genotype ON1 was detected in 247 (72.4%), NA1 in 92 (26.9%), and GA5 in 2 children (0.6%). RSV-A ON1, rarely observed in 2011/12, was the predominant RSV-A genotype in all settings by 2012/13 and remained predominant until 2016/17. Children in PP or PW infected with RSV-A ON1 did not show a more severe clinical course of disease compared with RSV-A non-ON1 infections. In the PICU group, hospital stay was one day longer (median 8 days, inter-quartile range (IQR) 7–12 vs. 7 days, IQR 5–9; *p* = 0.02) and duration of oxygen treatment two days longer (median 6 days, IQR 4–9 vs. 4 days, IQR 2–6; *p* = 0.03) for children infected with RSV-A ON1.

**Conclusions:**

In children, RSV-A ON1 largely replaced RSV-A non-ON1 genotypes within two seasons and remained the predominant RSV-A genotype in Germany during subsequent seasons. A higher clinical severity of RSV-A ON1 was observed within the group of children receiving PICU treatment, whereas in other settings clinical severity of RSV-A ON1 and non-ON1 genotypes was largely similar.

**Electronic supplementary material:**

The online version of this article (10.1186/s12879-019-4266-y) contains supplementary material, which is available to authorized users.

## Background

Human Respiratory Syncytial Virus (RSV) remains one of the leading causes of pediatric acute respiratory tract infections (RTI) affecting virtually all children by the age of two years [1–3]. In 2015, 33 million episodes of lower RTI due to RSV causing approximately 3 million hospital admissions and about 60,000 in-hospital deaths were estimated globally in children under 5 years of age [4]. The main clinical characteristics are cough, fever, dyspnea and wheezing [5]. Premature birth and young age are predisposing factors for a severe course of RSV disease and hospitalization due to RSV infection [2, 4–6].

Phylogenetically, RSV is divided into the major antigenic groups A and B, each with various genotypes, based mainly on genetic variability of the viral surface G glycoprotein [1, 7, 8]. In 2010, a novel RSV-A genotype ON1 characterized by a nucleotide (nt) duplication of 72-nt in the G protein gene, appeared in Ontario (Canada) [9]. A similar 60-nt duplication in the G protein gene of RSV-B genotype BA had previously been associated with rapid spread and replacement of other genotypes [10], indicating selection advantages of such nt-duplications [11, 12]. Correspondingly, it has been suggested that the new RSV-A ON1 may have the potential to replace other (non-ON1) RSV-A strains [2, 11, 13].

In Germany, RSV-A ON1 was first detected in 2011/12 [14], and was already predominant in the season 2012/13 [11]. Preliminary observations in hospitalized children in 2011/12 indicated a more severe clinical course of illness for RSV-A ON1 than for RSV-A non-ON1 genotypes [14], whereas a monocentric hospital study conducted during the season 2012/13 found no such difference [11].

In the multicenter investigation presented here, we analyzed the overall genotype distribution of RSV-A and RSV-B strains in out- and inpatient pediatric treatment settings in Germany during the period 2010/11 to 2016/17. The main objectives were to investigate the spread of the new genotype RSV-A ON1 and to assess its disease severity compared to RSV-A non-ON1 genotypes.

## Methods

### Study design

Data were obtained from ongoing surveillance studies on acute viral RTI conducted in the German Federal State Bavaria, including outpatients in pediatric practices (PP), inpatients hospitalized in a general pediatric ward (PW), and inpatients admitted to pediatric intensive care units (PICU). In all settings, children presenting with signs or symptoms of upper or lower RTI were included, and respiratory samples were initially tested for RSV infection and other respiratory viruses by multiplex PCR. Demographic and clinical patient data were obtained in all settings from standardized case report forms filled in by the treating physician (PP, PICU) or by the investigators based on the review of hospital patient files (PW). Detailed descriptions of the PP and PICU settings are to be found in previous publications on patients with other viral RTI [15, 16].

### Study population

PP setting: Children 1–5 years of age presenting with febrile RTI were prospectively enrolled at 33 PP in Bavaria during the years 2013 to 2015 (observation periods from January to May). Three children hospitalized during the course of disease were excluded from the PP setting.

PW setting: Children up to 16 years of age hospitalized due to RTI were retrospectively identified at a general PW (University Hospital of Würzburg, Bavaria); the observation period was January 2012 to March 2017 (all months). Case identification was based on the hospital laboratory database (PCR-confirmed RSV) and hospital patient files (admittance due to RTI). One child admitted to PICU during the course of disease was excluded from the PW setting.

PICU setting: a) Children 1 month to 16 years of age requiring intensive care treatment due to RTI were prospectively enrolled at 23 PICU in Bavaria from December 2010 to May 2013 (all months); b) Children 1 month to 5 years of age requiring intensive care treatment due to RTI were prospectively enrolled at 9 PICU in Bavaria during the years 2014 to 2017 (observation periods from January to May).

### Laboratory analyses

From all children included in the surveillance studies, nasopharyngeal, pharyngeal or throat swab samples or other respiratory secretions were obtained, during the initial practice visit at the PP or at the time of PW or PICU admittance, respectively. The samples were placed in a viral transport medium and analyzed at the Institute for Virology and Immunobiology, University of Würzburg. For the initial screening for RSV, a commercial multiplex PCR assay (“FTD Respiratory pathogens 21”, Fast Track Diagnostics Luxembourg S.à.r.l., Esch-sur-Alzette, Luxembourg) targeting RSV and other viral respiratory pathogens was used, in accordance with the manufacturer’s instructions. The assay does not discriminate between RSV-A and RSV-B. For RSV subtyping and genotyping, the second variable region of the G protein gene was amplified by RT-PCR using primers described by Peret et al. [7]. PCR fragments were sequenced in both directions using Big Dye terminator v3.1 chemistry and the ABI Prism 3130xl (Life Technologies, Darmstadt, Germany). RSV genotypes were assigned after alignment with reference sequences and subsequent phylogenetic analysis using MEGA software (Version MEGA 7.0.26). For this purpose, the best-fit nucleotide substitution model was calculated. Phylogenetic trees were constructed using the maximum likelihood algorithm and the General time-reversible model with a gamma distribution of 5. To test the tree stability, bootstrap values of 1,000 were used.

### Data management and statistical analysis

Data management was performed at the Department of Pediatrics, University Hospital of Würzburg. Data were entered twice into Microsoft Access databases and reconciled using Epi Info™ (CDC, Atlanta, USA) Version 3.3.2. Statistical analyses were carried out using IBM SPSS Statistics version 24.0 (IBM Corporation, New York, USA).

Only children with PCR-confirmed RSV infection and completed case report form were included in the analysis. Descriptions of seasonal and genotype distribution refer to children tested positively either for RSV-A or RSV-B, followed by genotyping. Comparison of clinical characteristics and severity within each setting were restricted to children with RSV-A infection, stratified by genotype (‘RSV-A ON1’ and ‘RSV-A non-ON1’). The RSV-A ON1 samples were further stratified by ‘early ON1’ (first two seasons of ON1 detections, 2011/12 and 2012/13) and ‘late ON1’ (season 2013/14 to 2016/17) for subgroup comparisons of severity within each setting. Children requiring PICU treatment were additionally stratified by premature birth status (yes/no).

Inter-setting differences between children admitted to PW or PICU were tentatively explored in a subgroup of children of the same age group (1 month to 5 years of age) hospitalized during the same seasons (‘early ON1’, 2011/12 or 2012/13) and months (January to May) in order to evaluate RSV-A ON1 as a potential risk factor for a more severe clinical course. In this subgroup, we analyzed whether hospitalized children infected with RSV-A who required intensive care treatment (PICU children) were more likely to be infected with the ON1 genotype than hospitalized children infected with RSV-A who did not require intensive care treatment (PW children).

Continuous variables are presented as median values and quartiles, and were compared between groups by using the Mann-Whitney U-test. Nominal variables were presented as absolute frequencies and percentages, and were compared between groups using Fisher’s exact test. For the inter-setting comparison of PW and PICU children regarding the RSV-A genotype, crude and adjusted odds ratios (OR) with corresponding 95% confidence intervals (95% CI) and *p*-values were calculated using logistic regression analyses; adjustment included the variables age, sex, season, premature birth status and ‘other pre-existing chronic conditions’ (including chronic lung disease, chronic neurological disorders, cardiac malformation, immunocompromising conditions, genetic disorders, and other chronic conditions reported by the treating physicians). Two-tailed p-values < 0.05 were considered statistically significant.

## Results

### RSV detection and overall genotype distribution

From 2010/11 to 2016/17, a total of 603 children with RTI due to PCR-confirmed RSV infection were included in the study (132 children from PP, 288 from PW, and 183 from PICU; the regional distribution of PP, PW and PICU contributing these patients is indicated in Additional file 1: Figure S1). Of these 603 children, 341 (56.6%) were infected solely with RSV-A and 235 (39.0%) solely with RSV-B. In 26 (4.3%) children, the RSV subtype could not be identified; these and one (0.2%) child co-infected with RSV-A and RSV-B were excluded from all subsequent analyses.

The seasonal distribution of RSV-A and RSV-B is shown in Table 1. RSV-A predominated during the first three and the last observed season, with the highest proportions observed in seasons 2011/12 (65%) and 2012/13 (81%). RSV-B predominated in the seasons 2013/14 to 2015/16.

**Table 1:** Seasonal distribution of *N* = 576 children with acute respiratory tract infection positively tested either for RSV-A or RSV-B. Samples were collected from pediatric practices (PP), a pediatric ward (PW), and/or pediatric intensive care units (PICU); Bavaria (Germany), 2010/11 to 2016/17

Season	All RSV	RSV-A	RSV-B	Setting
2010/11	50 (100)	26 (52.0)	24 (48.0)	PICU
2011/12	63 (100)	41 (65.1)	22 (34.9)	PW, PICU
2012/13	189 (100)	153 (81.0)	36 (19.0)	PP, PW, PICU
2013/14	71 (100)	29 (40.8)	42 (59.2)	PP, PW, PICU
2014/15	89 (100)	42 (47.2)	47 (52.8)	PP, PW, PICU
2015/16	67 (100)	25 (37.3)	42 (62.7)	PW, PICU
2016/17	47 (100)	25 (53.2)	22 (46.8)	PW, PICU
All seasons	576 (100)	341 (59.2)	235 (40.8)	PW, PICU

Data shown are numbers of cases (percent of all RSV cases, per season)

The genotypes detected in the 341 patients infected with RSV-A were ON1 (*n* = 247, 72.4%) and the non-ON1 genotypes NA1 (*n* = 92, 26.9%), and GA5 (n = 2, 0.6%). All 235 RSV-B detections were identified as genotype BA (100%); the sub-genotypes were BA9 (*n* = 154; 65.5%), BA4 (*n* = 61; 26.0%), BA10 (*n* = 16; 6.8%), BA2 (*n* = 3; 1.3%) and BA5 (n = 1; 0.4%).

### Spread of RSV-A ON1

**Fig. 1:**
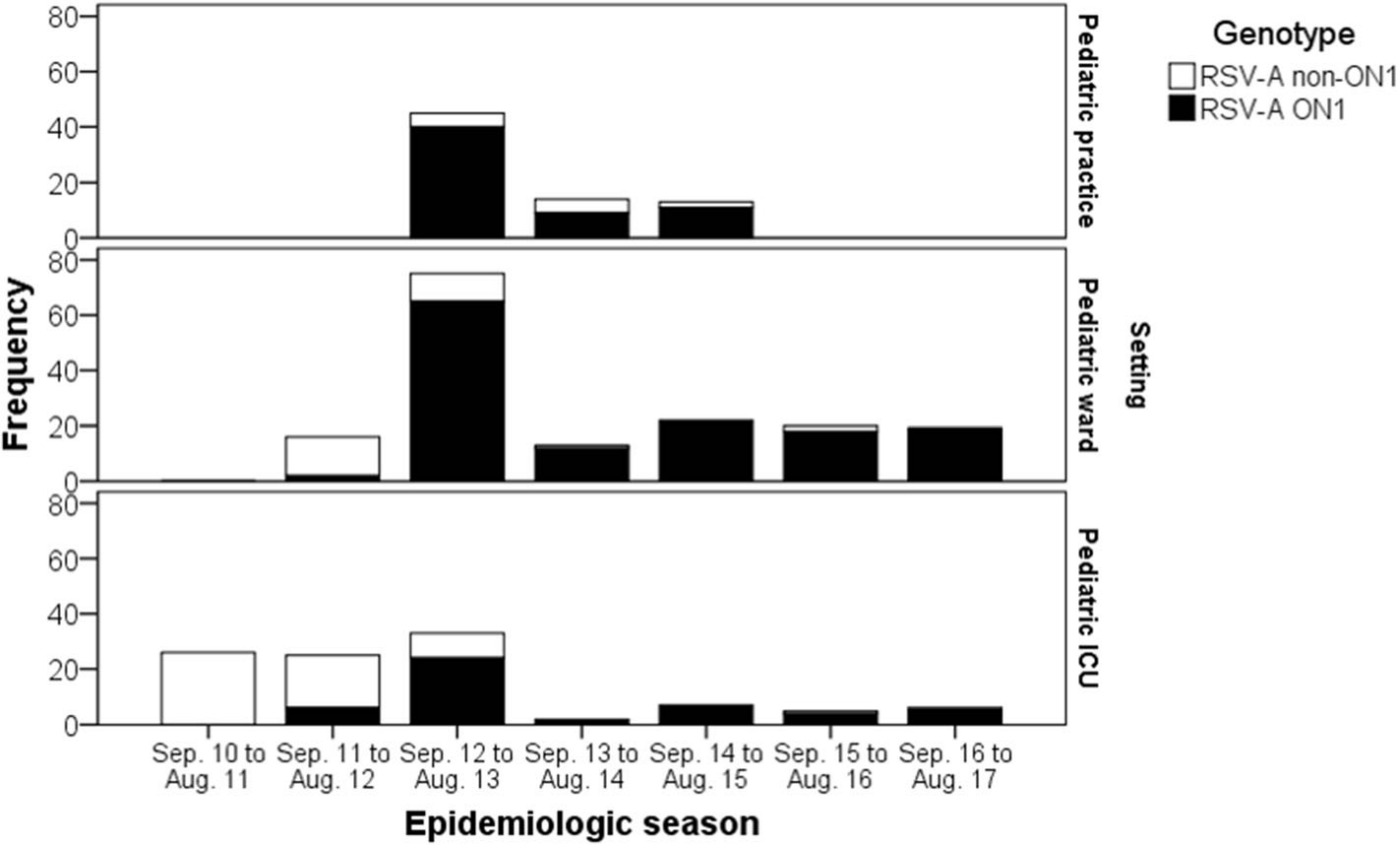
Seasonal distribution of 341 children with acute respiratory tract infection with RSV-A ON1 (*n* = 247) or RSV-A non-ON1 infection (*n* = 94) in pediatric practices (PP), a general pediatric ward (PW) and pediatric intensive care units (PICU) in Bavaria/Germany, 2010/11 to 2016/17. The columns represent the number of children included per RSV season. Note that any inter-setting comparison should be regarded with caution, as data were collected from separate surveillance studies differing in observation period, number of participating units, and inclusion criteria: in 33 PP, 72 children from 1 to 5 years of age with RSV-A infection were included during the RSV-seasons 2012/13 to 2014/15. In the PW, 165 children up to 16 years of age with RSV-A infection were included from 2011/12 to 2016/17. In 23 PICU, 104 children from 1 month to16 years of age with RSV-A infection were included from 2010/11 to 2012/13, and in 9 PICU, children from 1 month to 5 years of age were included during 2013/14 to 2016/17. The numbers per setting and RSV-A subtype are listed in Additional file 2: Table S1

Of all 341 RSV-A positive patients, 72 (21.1%) were cases reported in the PP surveillance study, 165 (48.4%) were cases identified in the PW surveillance and 104 (30.5%) were reported within the PICU surveillance studies. Following detection of the first cases of RSV-A ON1 in 2011/12, RSV-A ON1 spread rapidly and was found in all regions of Bavaria in 2012/13. The new genotype ON1largely replaced the RSV-A non-ON1 genotypes in all three settings, with the highest absolute number of genotype ON1 patients detected in the season 2012/13 in all settings (see Fig. 1 and Additional file 2: Table S1).

Outpatient data from PP was not available for analysis for the season 2011/12, the first season in which RSV-A ON1 was detected in Germany. RSV-A ON1 was present in 88.9% of all outpatient RSV-A samples reported for the season 2012/13, and remained the most prevalent RSV-A genotype in 2013/14 (64.3%) and 2014/15 (84.6%).

In the PW, RSV-A ON1 was present in 12.5% of all RSV-A infections in the season 2011/12 (start of data collection in January 2012). The proportion of RSV-A ON1 increased from 86.7% in 2012/13 to 92.3% in 2013/14 and to 100% in 2014/15. RSV-A ON1 remained the most prevalent RSV-A genotype until the end of the observational period (90% in 2015/16; 100% in 2016/17).

In the PICU setting, the proportion of genotype ON1 increased from 24.0% of all RSV-A samples detected in the first season with ON1 circulation (2011/12) to 72.7% in 2012/13 and to 100% in 2013/14. RSV-A ON1 remained the most prevalent RSV-A genotype until the end of the observational period (100% in 2014/15, 80% in 2015/16, 100% in 2016/17).

### Demographic and clinical characteristics of patients positive for RSV-A

Table 2 shows demographic details and clinical characteristics (including severity markers) of 341 children with RSV-A, stratified by pediatric treatment setting and genotype. Compared to patients infected with RSV-A non-ON1 genotypes, the clinical characteristics of patients with RSV-A ON1 infections appeared similar both in the PP setting (72 patients, 44.4% female, median age 2.5 years), and in the PW setting (165 patients, 43.0% female, median age 1.2 years). Of the 104 children treated in the PICU setting (45.2% female, median age 2.1 months), those infected with RSV-A ON1 tended to be older than children infected with RSV-A non-ON1 genotypes (median 3.4 months vs. 1.8 months; *p* = 0.02), and they had more often been born prematurely (34.7% vs. 16.4%, *p* = 0.04). In terms of clinical severity, they spent one day longer in hospital (median 8 days vs. 7 days; p = 0.02) and the duration of oxygen treatment was two days longer than for children with RSV-A non-ON1 genotypes (median 6 days vs. 4 days; *p* = 0.03). Additional subgroup analysis of PICU children stratified by premature/non-premature birth status (Table 3) confirmed the prolonged duration of hospital stay and oxygen treatment for RSV-A ON1 cases in the subgroup of non-prematurely born children, but not in the subgroup of prematurely born children. The difference observed in the duration of oxygen treatment remained significant when the analysis was further restricted to children < 6 months of age (*p* = 0.021; data not shown).

**Table 2:** Characteristics of N = 341 children with acute respiratory tract infection due to RSV-A, stratified by setting^a^ and RSV-A subtype (RSV-A ON1 vs. RSV-A non-ON1); Bavaria (Germany), 2010/11–2016/17

	Pediatric practice (PP)			Pediatric ward (PW)			Pediatric intensive care unit (PICU)		
	ON1	non-ON1	p	ON1	non-ON1	p	ON1	non-ON1	p
	*N* = 60 (100)	*N* = 12 (100)	–	*N* = 138 (100)	*N* = 27 (100)	–	N = 49 (100)	*N* = 55 (100)	–
Female sex	28 (46.7)	4 (33.3)	0.53	55 (39.9)	16 (59.3)	0.88	20 (40.8)	27 (49.1)	0.44
Age, years	2.5 (1.9; 3.7)	2.4 (1.9; 3.4)	0.75	1.1 (0.5; 2.3)	1.4 (0.6; 2.9)	0.50	0.3 (0.1; 1.5)	0.2 (0.1; 0.5)	**0.02**
Age, month	–	–	–	–	–	–	3.4 (1.7; 18.3)	1.8 (1.5; 6.4)	**0.02**
Premature birth	3 (5.0)	0 (0)	1.00	17 (12.3)	4 (14.8)	0.75	17 (34.7)	9 (16.4)	**0.04**
Other preexisting condition^b^	6 (10.0)	2 (16.7)	0.61	52 (37.7)	6 (22.2)	0.19	13 (26.5)	10 (18.2)	0.35
Viral coinfection^c^	29 (48.3)	7 (58.3)	0.75	46 (33.3)	12 (44.4)	0.28	22 (44.9)	16 (29.1)	0.11
Considered severe	1 (1.7)	1 (8.3)	0.31	–	–	–	–	–	–
Considered life threatening	–	–	–	–	–	–	7 (15.9)	10 (18.9)	0.79
Cough	48 (80.0)	9 (75.0)	0.71	107 (77.5)	27 (100)	**< 0.01**	41 (83.7)	44 (80.0)	0.80
Rhinitis	47 (78.3)	10 (83.3)	1.00	83 (60.1)	21 (77.8)	0.13	36 (73.5)	32 (58.2)	0.14
Refusal to eat / drink	5 (8.3)	1 (8.3)	1.00	65 (47.1)	17 (63.0)	0.15	25 (51.0)	35 (63.6)	0.24
Tachypnea	–	–	–	53 (38.4)	10 (37.0)	1.00	44 (89.8)	43 (78.2)	0.12
Fever	59 (100)	12 (100)	–	68 (49.3)	12 (44.4)	0.68	17 (34.7)	16 (29.1)	0.67
Fever, °C	39.8 (39.0; 40.2)	39.3 (39.1; 40.3)	0.91	38.7 (38.2; 39.3)	39.2 (38.5; 39.8)	0.07	38.8 (38.3; 39.0)	39.0 (38.8; 39.4)	0.20
Bronchitis / Bronchiolitis	26 (43.3)	4 (33.3)	0.75	68 (49.3)	9 (33.3)	0.15	37 (75.5)	47 (85.5)	0.22
Pneumonia	5 (8.3)	0 (0)	0.58	36 (26.1)	11 (40.7)	0.16	35 (71.4)	37 (67.3)	0.68
Sec. bacterial pneumonia	–	–	–	1 (0.7)	0 (0)	1.00	15 (30.6)	11 (20.0)	0.26
Symptoms, days	11 (8.5; 14)	10.5 (7; 13.5)	0.91	–	–	–	–	–	–
Hospital stay, days	–	–	–	3.0 (2.0; 5.0)	4.0 (3.0; 6.0)	0.10	8.0 (6.5; 12.0)	7.0 (5.0; 9.0)	**0.02**
PICU stay, days	–	–	–	–	–	–	5.0 (3.0; 7.5)	4.0 (2.0; 7.3)	0.23
Oxygen	–	–	–	59 (42.8)	12 (44.4)	1.00	47 (95.9)	46 (83.6)	0.06
Oxygen, days	–	–	–	3.0 (1.8; 5.0)	2.0 (1.8; 5.3)	0.77	6.0 (4.0; 9.0)	4.0 (2.0; 6.0)	**0.03**
CPAP	–	–	–	–	–	–	20 (40.8)	16 (29.1)	0.22
CPAP, days	–	–	–	–	–	–	2.5 (2.0; 4.8)	2.5 (2.0; 5.5)	0.89
Mechanical ventilation	–	–	–	–	–	–	7 (14.3)	4 (7.3)	0.34
Mechanical ventilation, days	–	–	–	–	–	–	6.5 (2.5, 8.5)	6.0 (2.3; 12.0)	0.91
Well-being at discharge	–	–	–	137 (99.3)	27 (100)	1.00	48 (98.0)	53 (96.4)	1.00
Well-being 14 days past visit	53 (89.8)	11 (91.7)	1.00	–	–	–	–	–	–

Data shown are number of cases (percent of N) or median (quartiles). P-values were derived using Fisher’s exact test or Mann-Whitney U-test, as appropriate. P-values in boldface indicate statistical significance

^a^Note that data from PP, PW and PICU were collected during different seasons (PP: 2012/13–2014/15, PW: 2011/12–2016/17, PICU: 2010/11–2016/17), and in different age groups (PP 1–5 years, PW and PICU up to 16 years)

^b^Including mainly chronic lung disease (11% of 341patients), chronic neurological disorders (9%), cardiac malformation (6%), and immunocompromising conditions (4%)

^c^Refers to coinfection with a different respiratory virus detected in the initial multiplex PCR

**Table 3:** Subgroup analyses of *N* = 104 children with respiratory tract infection due to RSV-A admitted to PICU, stratified by premature birth status; Bavaria (Germany) 2010/11–2016/17

	PICU All children			PICU Premature birth			PICU Non-premature birth		
	ON1	non-ON1	p	ON1	non-ON1	p	ON1	non-ON1	p
	N = 49 (100)	N = 55 (100)	–	*N* = 17 (100)	N = 9 (100)	–	*N* = 32 (100)	*N* = 46 (100)	–
Female sex	20 (40.8)	27 (49.1)	0.44	9 (52.9)	3 (33.3)	0.43	11 (34.4)	24 (52.2)	0.17
Age, month	3.4 (1.7; 18.3)	1.8 (1.5; 6.4)	**0.02**	3.4 (1.7; 23.6)	2.7 (1.7; 3.2)	0.39	3.6 (1.7; 18.4)	1.7 (1.4; 7.6)	0.05
Other preexisting condition^a^	13 (26.5)	10 (18.2)	0.35	5 (29.4)	1 (11.1)	0.38	8 (25.0)	9 (19.6)	0.59
Viral coinfection^b^	22 (44.9)	16 (29.1)	0.11	11 (64.7)	3 (33.3)	0.22	11 (34.4)	13 (28.3)	0.62
Hospital stay, days	8.0 (6.5; 12.0)	7.0 (5.0; 9.0)	**0.02**	8.0 (7.5; 14.0)	8.5 (7.3; 28.3)	0.66	8.0 (6.0; 12.0)	7.0 (5.0; 9.0)	**0.02**
PICU stay, days	5.0 (3.0; 7.5)	4.0 (2.0; 7.3)	0.23	3.0 (2.0; 8.5)	8.5 (3.3; 11.8)	0.27	5.0 (3.0; 6.0)	4.0 (2.0; 5.3)	0.06
Oxygen	47 (95.9)	46 (83.6)	0.06	17 (100)	6 (66.7)	**0.03**	30 (93.8)	40 (87.0)	0.46
Oxygen, days	6.0 (4.0; 9.0)	4.0 (2.0; 6.0)	**0.03**	5.0 (3.8; 9.0)	6.0 (4.0; 20.0)	0.35	6.0 (4.0; 9.0)	4.0 (2.0; 5.3)	**0.01**
CPAP	20 (40.8)	16 (29.1)	0.22	8 (47.0)	5 (55.6)	1.00	12 (37.5)	11 (23.9)	0.22
CPAP, days	2.5 (2.0; 4.8)	2.5 (2.0; 5.5)	0.89	3.0 (2.0; 4.5)	2.0 (2.0; 4.5)	0.70	2.0 (2.0; 4.8)	3.0 (2.0; 8.0)	0.66

Data shown are number of cases (percent of N) or median (quartiles). P-values were derived using Fisher’s exact test or Mann-Whitney U-test, as appropriate. P-values in boldface indicate statistical significance

^a^Included mainly chronic neurological disorders (14% of 104 patients), chronic lung disease (12%), cardiac malformations (7%), and genetic disorders (7%)

^b^Viral coinfections: 38 (37%) children with RSV-A were co-infected with at least one other respiratory virus, most frequently rhinovirus (*n* = 14) or coronavirus (n = 12)

In order to evaluate a potentially higher clinical severity of the new RSV-A ON1 genotype in children presumably immunologically naïve to this genotype, we additionally compared severity in children infected with RSV-A ON1 during the first two seasons of genotype ON1 appearance in Germany (‘early’, 2011/12 to 2012/13) with children infected with RSV-A ON1 during later ON1 seasons (2013/14 to 2016/17; Table 4). Within all three settings, clinical severity of RSV-A ON1 was similar for infections during the early and later seasons, except for a slightly longer duration of CPAP treatment in PICU children with early RSV-A ON1 (median 3.0 days vs. 2.0 days; *p* = 0.01).

**Table 4:** Subgroup analysis of N = 247 children with acute respiratory tract infection due to RSV-A ON1, stratified by setting and the first ON1 seasons (‘early’, 2011/12 to 2012/13) vs. later ON1 seasons (‘late’, 2013/14 to 2016/17); Bavaria (Germany), 2011/12–2016/17

	Pediatric practice (PP)			Pediatric ward (PW)			Pediatric intensive care unit (PICU)		
	Early ON1	Late ON1	p	Early ON1	Late ON1	p	Early ON1	Late ON1	p
	*N* = 40 (100)	*N* = 20 (100)		*N* = 67 (100)	*N* = 71 (100)	–	*N* = 30 (100)	*N* = 19 (100)	–
Female sex	22 (55.0)	6 (30.0)	0.10	27 (40.3)	28 (37.4)	1.00	16 (53.3)	4 (21.1)	**0.04**
Age, years	2.6 (1.8; 3.9)	2.5 (2.1; 3.6)	0.84	1.1 (0.5; 2.2)	1.1 (0.5; 2.3)	0.87	0.2 (0.1; 1.0)	0.5 (0.2; 1.7)	0.19
Age, month	–	–	–	–	–	–	2.3 (1.5; 12.8)	5.8 (2.1; 20.0)	0.19
Premature birth	1 (2.5)	2 (10.0)	0.26	7 (10.4)	10 (14.1)	0.61	11 (36.7)	6 (31.6)	0.77
Other preexisting condition	6 (15.0)	0 (0)	0.17	19 (28.4)	33 (46.5)	**0.04**	6 (20.0)	7 (36.8)	0.32
Viral coinfection	20 (50.0)	9 (45.0)	0.78	23 (34.3)	23 (32.4)	0.86	15 (50.0)	7 (36.8)	0.40
Considered severe	1 (2.5)	0 (0)	1.00	–	–	–	–	–	–
Considered life threatening	–	–	–	–	–	–	4 (13.3)	3 (21.4)	0.66
Cough	32 (80.0)	16 (80.0)	1.00	52 (77.6)	55 (77.5)	1.00	27 (90.0)	14 (73.7)	0.23
Rhinitis	29 (72.5)	18 (90.0)	0.19	43 (64.2)	40 (56.3)	0.39	25 (83.3)	11 (57.9)	**0.01**
Refusal to eat / drink	4 (10.0)	1 (5.0)	0.66	38 (56.7)	27 (38.0)	**0.04**	15 (50.0)	10 (52.6)	1.00
Tachypnea	–	–	–	21 (31.3)	32 (45.1)	0.12	26 (86.7)	18 (94.7)	0.64
Fever	39 (100)	20 (100)	–	32 (47.8)	36 (50.7)	0.74	10 (33.3)	7 (36.8)	1.00
Fever, °C	39.7 (39.0; 40.1)	40.0 (39.1; 40.2)	0.41	38.7 (38.2; 39.2)	38.8 (38.3; 39.4)	0.35	38.8 (38.5; 39.1)	38.8 (38.1; 39.6)	0.77
Bronchitis / Bronchiolitis	19 (47.5)	7 (35.0)	0.42	37 (55.2)	31 (43.7)	0.23	22 (73.3)	15 (78.9)	0.74
Pneumonia	4 (1.0)	1 (5.0)	0.66	17 (25.4)	19 (26.8)	1.00	19 (63.3)	16 (84.2)	0.19
Sec. bac.pneumonia	–	–	–	0 (0)	1 (1.4)	1.00	7 (23.3)	8 (42.1)	0.21
Symptoms, days	11.0 (9.0; 14.0)	10.0 (7.0; 15.0)	0.92	–	–	–	–	–	–
Hospital stay, days	–	–	–	3.0 (2.0; 5.0)	3.0 (2.0; 5.0)	0.68	8.0 (6.0; 11.3)	10.0 (7.0; 14.0)	0.21
PICU stay, days	–	–	–	–	–	–	4.5 (3.0; 6.3)	5.0 (2.0; 10.0)	0.76
Oxygen	–	–	–	26 (38.8)	33 (46.5)	0.39	28 (93.3)	19 (100)	0.51
Oxygen, days	–	–	–	4.0 (3.3; 4.8)	3.0 (1.0; 5.0)	0.32	5.0 (4.0; 7.0)	8.5 (4.3; 14.3)	0.06
CPAP	–	–	–	0 (0)	0 (0)	–	15 (50.0)	5 (26.3)	0.14
CPAP, days	–	–	–	–	–	–	3.0 (2.0; 5.0)	2.0 (1.0; 2.0)	**0.01**
Mechanical ventilation	–	–	–	0 (0)	0 (0)	–	4 (13.3)	3 (15.8)	1.00
Mechanical ventilation, days	–	–	–	–	–	–	3.0 (1.0; −)	7.0 (6.0; −)	0.18
Well-being at discharge	–	–	–	67 (100)	70 (98.6)	1.00	30 (100)	18 (94.7)	0.39
Well-being 14 days past visit	33 (90.0)	17 (89.5)	1.00	–	–	–	–	–	–

Data shown are number of cases (percent of N) or median (quartiles). P-values were derived using Fisher’s exact test or Mann-Whitney U-test, as appropriate. P-values in boldface indicate statistical significance

To further evaluate a potential association of RSV-A genotype and a more severe course of RSV-A disease, a pooled subgroup of hospitalized children < 6 years of age admitted with RSV-A either to PW (*n* = 80) or PICU (*n* = 49) during the same months of the seasons 2011/12 or 2012/13 was explored. The proportion of children infected with RSV-A ON1 was 81.3% (61 of 80 children) for patients treated in PW, whereas this proportion was 57.1% (28 of 49 children) for patients treated in PICU. Unadjusted, logistic regression analysis indicated RSV-A ON1 infections being associated less frequently with PICU treatment than infections with other RSV-A genotypes (OR 0.42, 95% CI 0.19–0.89; *p* = 0.02). However, after adjustment for sex, age, season, premature birth and other preexisting conditions, RSV-A ON1 infection was no longer significantly associated with less frequent PICU treatment (OR 0.52, 95% CI 0.18–1.51; *p* = 0.64).

## Discussion

After its first detection in Germany in 2011/12, RSV-A ON1 co-circulated with RSV-A non ON1 genotypes and had largely replaced RSV-A non-ON1 genotypes in 2013/14, thus demonstrating a very rapid and effective spread. Following the first reports of RSV-A ON1 in Canada [9] and Germany [14], genotype ON1 was detected in many other countries and regions [3, 13], including Asia [8, 17–28], Africa [12, 29–31], Europe [32–39] and North and South America [40–43]. In line with our results, several multi-season studies have previously reported ON1 to have rapidly replaced RSV-A non-ON1 genotypes [18, 19, 25, 28, 30–33, 35, 37–39, 42–45]. Furthermore, ON1 has recently been reported to be the currently most prevalent RSV-A genotype worldwide [43].

During the first and the second season of RSV-A ON1 appearance in Germany, the proportion of RSV-A in our study population was considerably higher than the proportion of RSV-B, similar to reports from some other countries [19, 38, 43, 44]. It has been speculated that the rapid spread of RSV-A ON1 might also reduce RSV-B circulation in subsequent seasons [43, 44]. In our study, however, RSV-B strongly dominated the seasons 2013/14 and 2015/16. This finding suggests that the emergence of genotype ON1 has not had a noticeable effect on the overall epidemiological pattern of frequent shifts between RSV-A and RSV-B group dominance [2, 3], confirming similar observations reported from Spain [38] and Argentina [43].

The genotypes currently most prevalent worldwide (RSV-A ON1 and RSV-B BA) are both characterized by extensive nt-duplications in the G-gene. The mechanisms for the selective advantage of such nt-duplications are still poorly understood and may be associated with effects on virus replication fitness [46], or on the modulation or evasion of host immune response and host adaptation [3, 19]. It will be interesting to observe for how long these two genotypes remain predominant. For RSV-B genotype BA, first detected in 1999 [10], a considerable number of sub-genotypes based on point mutations are already known [3]. For RSV-A ON1, several new lineages have recently emerged [13], including the extreme case of a complete loss of the nt-duplication which has been observed under specific conditions, e.g. a lack of host’s immune system driven selective pressure [47]. The future evolution of these two most prevalent genotypes and the underlying selection mechanisms may have a considerable impact on the development of potential RSV vaccines [19].

Reports on the clinical disease severity of RSV-A ON1 compared to RSV-A non-ON1 genotypes are inconsistent and have thus far mainly been based on observations from inpatients [2]. Our investigation is one of only few studies describing RSV-A ON1 separately for children requiring outpatient primary care [25], and indicates similar disease severity for outpatients infected with RSV-A ON1 and those with RSV-A non-ON1 infections. Regarding inpatients, we also found a similar clinical severity of RSV-A genotypes in children admitted to a general PW. Interestingly, however, in the group of children requiring PICU treatment, our study suggested a somewhat more severe course of disease in children with genotype ON1 infection, such as a longer duration of hospital stay and of oxygen treatment, compared to children infected with RSV-A non-ON1.

Some of previous inpatient studies comparing RSV-A ON1 with RSV-A non-ON1 genotypes found no difference in clinical severity [11, 38], while others showed divergent correlations between genotype ON1 and viral virulence [2]. Several authors have suggested a higher virulence of RSV-A ON1, based on the observation of higher rates of PICU admission and/or mechanical ventilation [42], earlier hospitalization and a higher rate of lower respiratory tract infections [19] or more frequent refusal to feed [44]. In contrast, three studies reported a milder course of illness associated with genotype ON1, such as a lower severity score [32], a lower rate of bronchopneumonia / lower respiratory tract disease [33, 35] and a lower hospitalization rate [33]. These controversial observations might be explained by the heterogeneity of study designs, inclusion criteria, region, and the seasons/years when the surveillance was undertaken, resulting in different levels of genotype-specific herd immunity [2, 19, 44].

In populations immunologically naïve to a new genotype, an initially more severe clinical course of disease might be expected. A study conducted during the early phase of RSV-A ON1 spread in Vietnam indicated a higher hospitalization rate for genotype ON1 [19]. Furthermore, a study from Italy covering the first two years of genotype ON1 emergence [35] and also our own observations from the season 2011/12, the first year of genotype ON1 appearance in Germany [14], had suggested a higher rate of PICU admissions for RSV-A ON1 patients. However, our previous assumption was based solely on laboratory samples; clinical data to adjust for other factors with a potential impact on PICU admission was not available at that time [14]. The inter-setting analysis presented here was based on both laboratory and clinical data for a larger and more homogenous subgroup of PW and PICU patients admitted during the first two seasons of RSV-A ON1 emergence. In this analysis, we found no association of genotype ON1 with PICU treatment after adjustment for season, demographic and anamnestic characteristics. Furthermore, only very few of our within-setting comparisons pointed to a more severe clinical course of RSV-A ON1 during the early phase of ON1 circulation compared to later seasons, e.g. an increased refusal to feed in PW patients and longer CPAP treatment in PICU patients. Our study therefore showed no indication of a notably higher virulence of RSV-A ON1 during the first season(s) after its initial emergence.

The main difference between RSV-A genotypes observed in our analyses was the higher clinical severity in the group of PICU children infected with genotype ON1, compared to PICU children with RSV-A non-ON1 infection. The majority of children in this setting were younger than 6 months of age (median age two months), and likely experienced their first-ever RSV infection. RSV-A NA1 was the most prevalent RSV-A genotype in the population before the emergence of genotype ON1 and, hence, it might be speculated that maternal RSV-neutralizing antibodies transferred to the child before birth [48] were mainly directed against the NA1 genotype. As the mean half-life of RSV-neutralizing antibodies has been estimated as 26 days, it seems plausible that they may have provided at least partial immunity against the NA1 genotype during the first few months of life [48–50], but no or only very limited protection against the new ON1 genotype [51].

Our study has several limitations. The data for the present analyses was obtained within the framework of several ongoing surveillance studies, each targeting clinical severity of respiratory viruses in a specific pediatric setting. Hence, although all studies included children with PCR-confirmed, RSV-associated RTI and enabled extensive within-setting comparisons, the scope for inter-setting comparison was limited, mainly due to the differences in design, study seasons, and patient age range. Even in a subgroup corrected for study seasons and age range, the results of the inter-setting comparison PW vs. PICU should still be regarded with caution, as the children from PW and from PICU pooled for this analysis belonged to different study populations, with only one hospital contributing to the PW dataset. Hence, regional factors, such as possible differences in the distribution of RSV-A genotypes, cannot be ruled out, which may lead to confounding of the association. Furthermore, some subgroup analyses within settings were limited by the small sample size, e.g. the comparison of RSV-A genotypes in the subgroup of PICU children with premature birth. However, the higher clinical severity of genotype ON1 found in patients within the PICU setting was indicated by several markers of severity, both overall and in stratified analyses, and, thus, appears to be a robust finding. Apart from small sample size, the less pronounced difference among RSV-A genotypes observed in PICU children with premature birth might be due to the fact that the general higher risk for a more severe course of RSV disease of these children has a stronger impact than variations in the RSV genotype.

The main strengths of our study are the collection of a largely comprehensive set of clinical and laboratory data from three different pediatric settings reflecting various degrees of clinical severity. In particular, our study includes an evaluation of RSV-A ON1-associated clinical severity in primary care outpatients, who represent the majority of medically treated RSV cases, and a separate evaluation of genotype ON1-associated severity in pediatric PICU patients.

## Conclusion

In children, RSV-A ON1 largely replaced RSV-A non-ON1 genotypes in Germany within two epidemiological seasons and remained the prevalent RSV-A genotype during the following four seasons. Clinical severity of ON1 infections was similar to that of RSV-A non-ON1 infections both in out- and inpatient pediatric settings, apart from a longer treatment duration for ON1 patients within the group of children requiring PICU treatment.

## Additional files

Additional file 1: **Figure S1** Surveillance of RSV epidemiology in different settings in the Federal State Bavaria (Germany), 2010–2017. Study sites in Bavaria contributing RSV patients to the study: pediatric practices (PP), pediatric ward (PW), pediatric intensive care units (PICU). Study sites who enrolled patients with acute respiratory tract infection but without laboratory confirmation for RSV are not shown. (DOCX 63 kb)

Additional file 2: **Table S1** Seasonal distribution of *N* = 341 children with acute respiratory tract infection due to RSV-A. Data stratified by setting and RSV-A subtype (RSV-A ON1 vs. non-ON1 RSV-A). (DOCX 50 kb)

## Data Availability

The datasets generated and/or analyzed in this study are not publicly available as the surveillance studies in which the original data was collected included guarantees towards all participating practices, hospitals and patients, by contract or informed consent, that all patient data would be analyzed solely at the Department of Pediatrics, University Hospital of Würzburg, and would not be forwarded to any third party. Fully anonymized data will be available from the corresponding author upon reasonable request, as far as permitted by the Data Protection Office and the Legal Department of the University Hospital of Würzburg.

## Abbreviations

CI: Confidence interval
CPAP: Continuous positive airway pressure
G: Glycoprotein
IQR: Interquartile range
mo.: Months
nt: Nucleotides
OR: Odds ratio
p: *p*-value
PCR: Polymerase chain reaction
PICU: Pediatric intensive care unit(s)
PP: Pediatric practice(s)
PW: Pediatric ward(s)
RSV: Respiratory Syncytial Virus
RTI: Respiratory Tract Infection
