# Supplementary material for: Spread and clinical severity of respiratory syncytial virus A genotype ON1 in Germany, 2011–2017
Source: BMC Infect Dis. 2019 Jul 12;19:613. doi: 10.1186/s12879-019-4266-y (PMC6624929; doi:10.1186/s12879-019-4266-y)

**Additional file 1: Figure S1**

**Surveillance of RSV epidemiology in different settings in the Federal State Bavaria (Germany), 2010-2017**

Study sites in Bavaria contributing RSV patients to the study: pediatric practices (PP), pediatric ward (PW), pediatric intensive care units (PICU). Study sites who enrolled patients with acute respiratory tract infection but without laboratory confirmation for RSV are not shown.

Outline map from Bavaria and Bavarian districts: <https://d-maps.com/m/europa/germany/baviere/baviere52.gif> ; modified.


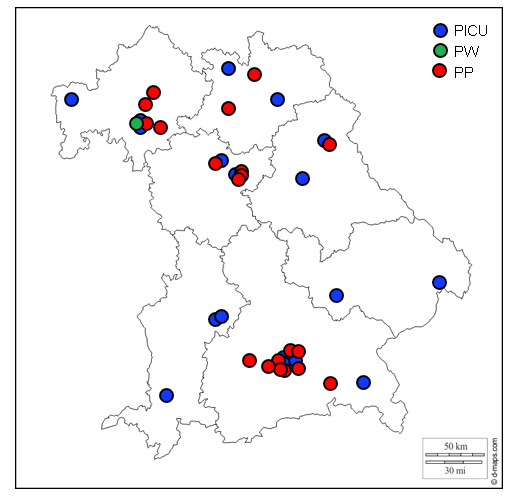

Supplement: Supplementary file 1 — Figure S1 Surveillance of RSV epidemiology in different settings in the Federal State Bavaria (Germany), 2010–2017. Study sites in Bavaria contributing RSV patients to the study: pediatric practices (PP), pediatric ward (PW), pediatric intensive care units (PICU). Study sites who enrolled patients with acute respiratory tract infection but without laboratory confirmation for RSV are not shown. (DOCX 63 kb) [file 12879_2019_4266_MOESM1_ESM.docx]
