# Supplementary material for: Spread and clinical severity of respiratory syncytial virus A genotype ON1 in Germany, 2011–2017
Source: BMC Infect Dis. 2019 Jul 12;19:613. doi: 10.1186/s12879-019-4266-y (PMC6624929; doi:10.1186/s12879-019-4266-y)
Supplement: Supplementary file 2 — Table S1 Seasonal distribution of N = 341 children with acute respiratory tract infection due to RSV-A. Data stratified by setting and RSV-A subtype (RSV-A ON1 vs. non-ON1 RSV-A). (DOCX 50 kb) [file 12879_2019_4266_MOESM2_ESM.docx]

**Additional file 2: Table S1:** Seasonal distribution of N=341 children with acute respiratory tract infection due to RSV-A. Data stratified by setting and RSV-A subtype (RSV-A ON1 vs. non-ON1 RSV-A)

| **Season** | **Pediatric practice (PP)** | | **Pediatric ward ( PW)** | | **Pediatric intensive care unit (PICU)** | |
| --- | --- | --- | --- | --- | --- | --- |
|  | **(n=72)** | | **(n=165)** | | **(n=104)** | |
|  | **ON1** | **Non-ON1** | **ON1** | **Non-ON1** | **ON1** | **Non-ON1** |
| 2010/11 | - | - | - | - | 0 (0.0) | 26 (100.0) |
| 2011/12 | - | - | 2 (12.5) | 14 (87.5) | 6 (24.0) | 19 (76.0) |
| 2012/13 | 40 (88.9) | 5 (11.1) | 65 (86.7) | 10 (13.3) | 24 (72.7) | 9 (27.3) |
| 2013/14 | 9 (64.3) | 5 (35.7) | 12 (92.3) | 1 (7.7) | 2 (100.0) | 0 (0.0) |
| 2014/15 | 11 (84.6) | 2 (15.4) | 22 (100.0) | 0 (0.0) | 7 (100.0) | 0 (0.0) |
| 2015/16 | - | - | 18 (90.0) | 2 (10.0) | 4 (80.0) | 1 (20.0) |
| 2016/17 | - | - | 19 (100.0) | 0 (0.0) | 6 (100.0) | 0 (0.0) |
